# Male breast cancer: experience from a quaternary hospital in Tanzania

**DOI:** 10.11604/pamj.2026.53.149.47743

**Published:** 2026-04-02

**Authors:** Nashivai Kivuyo, Mungeni Misidai, Daniel Kitua, Alfred Chibwae, Rajabu Bakari, Larry Akoko

**Affiliations:** 1 Department of Surgery, Muhimbili University of Health and Allied Sciences, Dar es Salaam, Tanzania,; 2 Department of General Surgery, Muhimbili National Hospital, Dar es Salaam, Tanzania

**Keywords:** Male breast cancer, male breast malignancy, Africa, male breast cancer survival, treatment outcomes

## Abstract

**Introduction:**

male breast cancer (MBC) is rare, but its incidence is increasing globally. In sub-Saharan Africa, breast cancer care has primarily focused on women, creating a gap in understanding MBC outcomes. This study aimed to examine the clinical characteristics and treatment outcomes of MBC patients at Tanzania´s National Hospital.

**Methods:**

this retrospective cohort study, conducted at Muhimbili National Hospital, included male patients aged 18 years and above with histologically confirmed breast cancer diagnosed between January 2018 and December 2024. Demographic and clinical data were extracted from treatment records and presented as frequencies, proportions, and means. Survival status, obtained from case notes or patient/next-of-kin interviews, was analyzed using the Kaplan-Meier method.

**Results:**

fifty-six MBC patients were identified, with a mean age of 60.6 ± 12.9 years. Most patients (71.1%) had T4 disease, and 26.8% had metastasis, primarily pulmonary (73%). Invasive ductal carcinoma accounted for 83.9% of cases. The most common molecular subtype was Luminal A (61.8%), followed by triple negative (20%). Nearly all patients (97%) underwent surgery, with modified radical mastectomy and simple mastectomy performed in 48% and 45%, respectively. Over 60% of patients treated with curative intent had recurrence within 15 months. The 5-year overall survival rate was 11.2%.

**Conclusion:**

advanced disease presentation was common among MBC patients, corresponding to poor overall survival. Additionally, early disease progression was noted among those treated with curative intent. These findings underscore gaps in healthcare-seeking behavior, screening, and referral systems. Addressing these challenges and optimizing treatment protocols are crucial for improving outcomes.

## Introduction

Male breast cancer (MBC) is a rare condition worldwide, accounting for approximately 1% of all breast cancer and overall male cancer diagnoses [1]. Although uncommon, its incidence has risen by about 26% over the past 25 years [2,3]. While MBC shares several characteristics with female breast cancer (FBC), key differences exist, particularly in age at diagnosis, hormone receptor expression, prognosis, and survival outcomes [4]. MBC is typically diagnosed at an older age and more advanced stage, contributing to poorer survival rates [5]. Additionally, MBC exhibits a distinct genetic profile, with fewer shared genetic drivers compared to FBC [6]. Recent studies have further identified two novel MBC subgroups, Luminal M1 and M2, which are entirely distinct from those seen in FBC [7]. These findings suggest that MBC may require a tailored approach to diagnosis and treatment, highlighting the need for further research into its unique biological and clinical characteristics.

A review of a hospital-based cancer registry from Tanzania conducted over a decade ago indicated that MBC accounted for approximately 6% of all BC cases [8]. However, this figure may have been overestimated due to referral bias, as the data were derived from a specialized cancer institute that primarily receives patients needing advanced care, such as radiation therapy, a common necessity for MBC, which often presents at later stages. Despite advancements in breast cancer management, there remains a significant gap in current clinical and epidemiological data on MBC, both in Tanzania and throughout sub-Saharan Africa. This lack of updated evidence hinders efforts to optimize care pathways for patients affected by this condition. To address this gap, the present study aimed to examine the clinical characteristics of MBC patients treated at a quaternary hospital in Tanzania.

## Methods

**Study design and setting:** we conducted a retrospective cohort study of male breast cancer cases. The reporting of this study follows the STROBE (Strengthening the Reporting of Observational Studies in Epidemiology) guidelines for cohort and cross-sectional studies.

**Setting:** the study was conducted at Muhimbili National Hospital (MNH) in Dar es Salaam, Tanzania. MNH is a quaternary-level teaching hospital that provides specialized oncology services for the country. We included cases diagnosed between January 1^st^ 2018, and December 31^st^, 2024.

**Participants:** we included all male patients, 18 years and above, with a histologically confirmed diagnosis of breast cancer diagnosed during the study period. We searched the electronic medical records (EMRs) of MNH to identify all patients with an ICD-10 code for breast cancer, then filtered the data to include only male patients. Their medical records were reviewed to ensure the presence of complete histopathological data, and cases lacking histopathological confirmation were excluded. Among the patients with available results, those with benign diagnoses were further excluded. To assess survival status, we contacted patients or their listed next of kin using the available contact information.

**Variables:** the variables abstracted included the patient's age, calculated from the date of birth to the date of histological diagnosis, and the documented stage of the disease at the time of diagnosis. Tumor histological type and hormonal receptor status (estrogen receptor (ER), progesterone receptor (PR), and HER2/Neu receptor status) were obtained from signed histology reports from the pathology laboratory. Additional variables included details of the treatment received. For patients who underwent surgery, we recorded the type of surgical procedure documented in surgical notes and the resection margin status reported in pathology records. Data on treatment intent were also collected, and overall survival and disease-free survival were assessed.

**Data sources/measurement:** clinical information was abstracted from patient medical records, oncology clinic notes, pathology reports, and the hospital cancer registry. Data abstraction was performed by trained clinicians using a standardized data collection form. Any discrepancies were resolved by consensus among investigators.

**Bias:** given the retrospective nature, data completeness and selection bias are possible. Some patient records were incomplete, introducing potential attrition bias. We attempted to minimize bias by including all eligible patients and systematically extracting available information. Two data abstractors were employed to extract study-related variables using a predefined study questionnaire. The two completed abstraction sheets were compared for each patient, and in cases of disagreement, either of the authors, NK or LA, would repeat the abstraction to resolve the discrepancy.

**Study size:** all consecutive male breast cancer cases meeting inclusion criteria during the 7-year period were included. A total of 56 patients fulfilled the criteria and were analyzed.

**Quantitative variables:** continuous variables are reported as means with standard deviations. Categorical variables are presented as counts and percentages.

**Statistical methods:** after the data were complete and verified, de-identification was conducted by removing patients´ names and hospital registration numbers from the Excel sheets. The data were then transferred to the Statistical Package for the Social Sciences (SPSS) version 27 for analysis. Categorical data were summarized as frequencies with proportions, while continuous variables were summarized as means and standard deviations. Kaplan-Meier survival analysis curves were plotted. Overall survival (OS) was analyzed for all patients. In the sub-analysis of disease-free survival (DFS), we only included patients with non-metastatic breast cancer who underwent a modified radical mastectomy for curative intent.

**Ethics approval:** the study protocol was reviewed and approved by the Institutional Review Board of MNH, with approval number MNH/IRB/I/052 and IRB extension number MNH/IRB/EXT/2025/002. To ensure patient confidentiality, all data were de-identified before analysis, and no direct patient identifiers were used. Given the retrospective nature of the study, the requirement for written informed consent was waived. However, oral consent was obtained from patients or their next of kin when contacted for survival outcome assessment.

## Results

**Figure 1:**
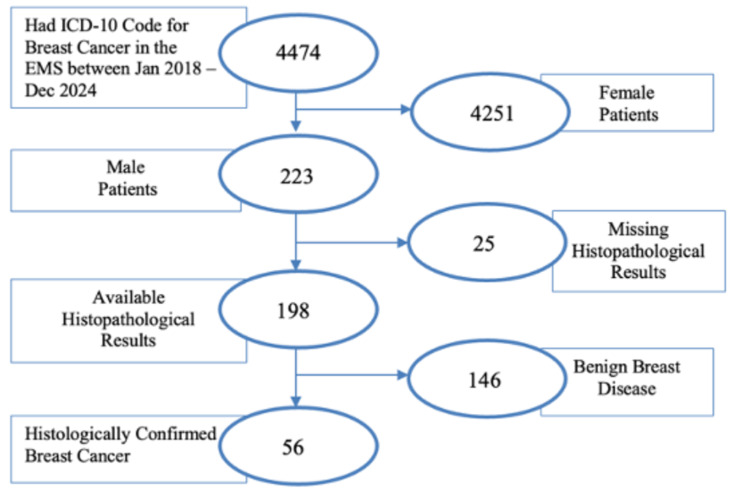
flow chart showing recruitment of male breast cancer patients treated at Muhimbili National Hospital between 2018 and 2024

Initially, 4,474 patients had an ICD-10 code for breast cancer recorded in the Emergency Medical Services (EMS) from January 2018 to December 2024. Of these, 4,251 were female patients, and 223 were male patients. Among the male patients, 198 had available histopathological results after excluding 25 with missing results. After further analysis, 146 patients were diagnosed with benign breast disease, while 56 were histologically confirmed as having MBC. The recruitment of study participants is illustrated in Figure 1.

**Clinicodemographic characteristics of MBC patients:** the mean age at diagnosis was 60.6 ± 12.9 years (range: 37-90), with the majority (18, 32.1%) in the 60-69 age group. Primary tumor status was documented in 38 patients, with the majority (71.1%) having T4 disease. T3 and T2 disease were each observed in 5 patients (13.4%), and only one patient (2.6%) had T1 disease. Regarding nodal involvement, data were available for 32 patients, with most (13, 40.6%) classified as N1, followed by N0 (34.4%). Distant metastases were identified in 15 patients (26.8%), with pulmonary metastases being the most common (73%). In terms of disease stage, most patients were diagnosed at advanced stages, with 20 (47.6%) in stage III and 15 (35.7%) in stage IV. Histologically, invasive ductal carcinoma (NOS) was the predominant type, accounting for 47 cases (3.9%). Among molecular subtypes, Luminal A was the most prevalent (21, 61.8%), followed by triple-negative (7, 20%), as seen in Table 1.

**Table 1:** clinicopathological characteristics of male breast cancer patients treated at Muhimbili National Hospital between 2018 and 2024

Variables	Frequency (%)
**Age**	
<40	1 (1.8)
40 - 49	11 (19.6)
50 - 59	14 (25.0)
60 - 69	18 (32.1)
70 - 79	7 (12.5)
>79	5 (8.9)
**T-status (N=38)**	
T1	1 (2.6)
T2	5 (13.4)
T3	5 (13.4)
T4	27 (71.1)
**N-status (N=32)**	
N0	11 (34.4)
N1	13 (40.6)
N2	6 (18.8)
N3	2 (6.3)
**TNM staging (N=42)**	
I	4 (9.5)
II	3 (7.1)
III	20 (47.6)
IV	15 (35.7)
**Site of metastasis (N=15)**	
Pulmonary	11 (73)
Spine	2 (13.3)
Liver	2 (6.7)
Brain	2(6.7)
**Histology (N=56)**	
Invasive ductal carcinoma NOS	47 (83.9)
Papillary carcinoma	5 (8.9)
Mucinous carcinoma	2 (3.6)
Lobular carcinoma	2 (3.6)
**Molecular subtype (N=34)**	
Luminal A	21 (61.8)
Triple negative	7 (20.6)
Luminal B	3 (8.8)
HER2 enriched	3 (8.8)

NOS: not otherwise specified; HER2: human epidermal growth factor receptor 2

**Figure 2:**
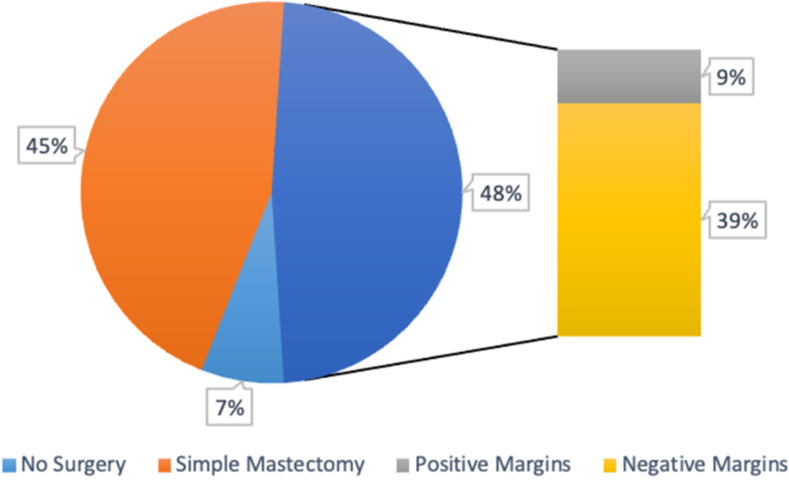
pie chart showing oncological outcomes among 27 MBC patients treated with curative intent

**Surgical treatment of MBC:** among the 56 MBC patients, 52(92.9%) had surgery, of which modified radical mastectomy (MRM) and simple mastectomy (mastectomy without lymph node dissection) were offered to 48% and 45%, respectively. Amongst those who had an MRM, 19% had positive resection margins as shown in Figure 2.

**Figure 3:**
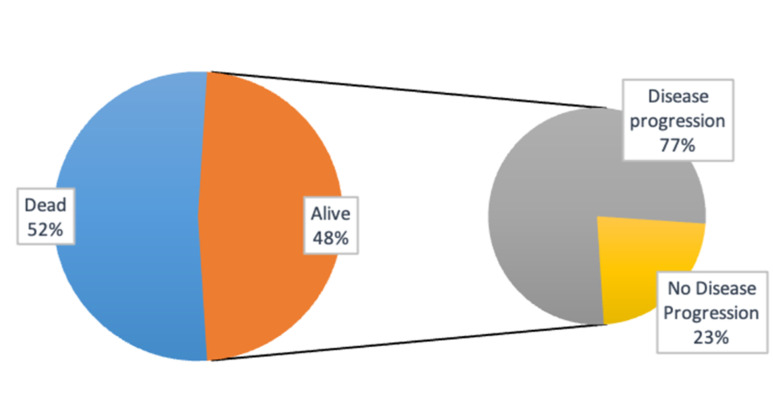
pie chart showing surgical treatments for MBC patients attended at MNH between 2018 and 2024

**Surgical and oncological outcomes:** patients were followed up for varying periods of time, ranging from 3 to 74 months, with a mean follow-up time of 38.5 months. During this follow-up, 52% of the patients had died, leaving 48% alive. Among those alive, 23% remained alive without documented disease progression, while 77% were living with disease progression, as shown in Figure 3.

**Figure 4:**
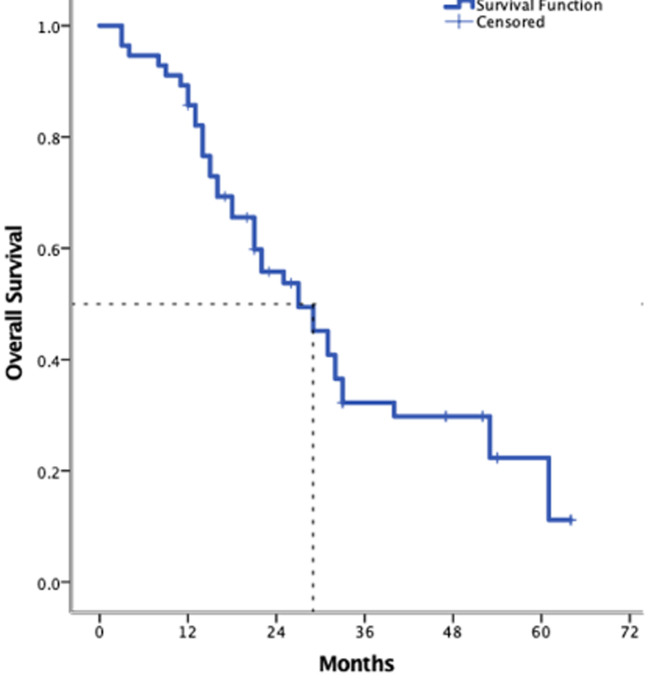
Kaplan-Meier curve showing overall survival among MBC patients attended at MNH between 2018-2024 (N=56)

**Overall survival of MBC patients attended at MNH:** regarding overall survival, patients experience significant declines in the first three years following diagnosis, indicating a notably higher mortality rate during this time, with more than half of the patients dying within the initial 27 months of follow-up. The 5-year survival probability, as estimated by the Kaplan-Meier curve in Figure 4, is 11.2%.

**Figure 5:**
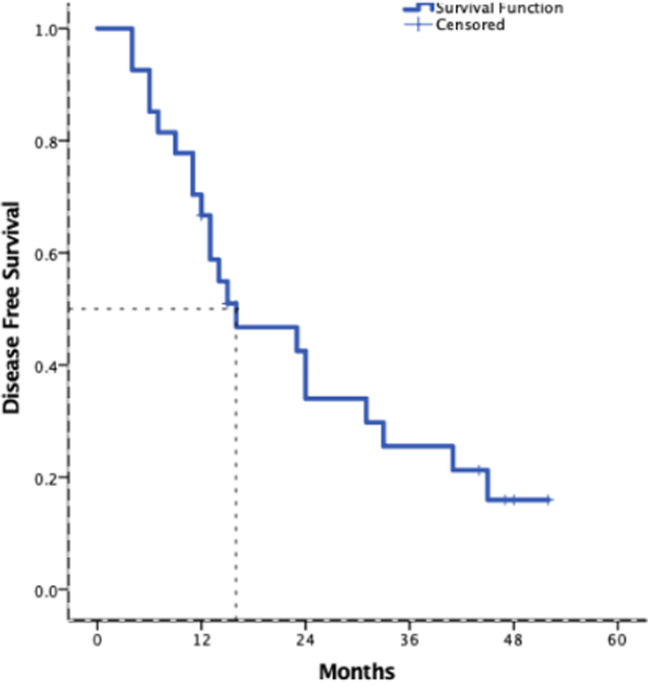
Kaplan-Meier curve showing disease-free survival among male breast cancer patients treated with curative intent at Muhimbili National Hospital between 2018 and 2024 (N=27)

**Disease-free survival of MBC patients treated with a curative intent at MNH:** the median DFS was 15.1 months. The curve shows a steady decline, with more than 60% of patients experiencing disease recurrence within 15 months of surgery. Figure 5 shows the Kaplan-Meier survival curves illustrating the DFS of the 27 MBC patients treated with curative intent at MNH. Beyond 48 months, the DFS probability is estimated at 16.7% or lower.

## Discussion

To the best of our knowledge, this is the first study from Tanzania to highlight the key clinical and pathological characteristics of MBC. The clinical and demographic characteristics reported align with global patterns while also suggesting potential areas for further investigation and intervention. This study failed to provide insight into the incidence of MBC to FBC due to the inability to review all histology reports of FBC cases. The mean age at diagnosis was 60.6 years, consistent with a recent retrospective review of pathology results in Northern Uganda [9]. Another similarity was the wide age variability among our patients. These similarities agree with global data suggesting that MBC is primarily a disease of the elderly, although younger males are not entirely protected [10].

A concerning finding is that most cases present with advanced disease, with nearly half in clinical stage III and about a third in stage IV. This late-stage presentation aligns with global trends in MBC, where late-stage diagnosis is common [11]. The high proportion of patients with nodal involvement (40.6% with N1) further highlights the serious concerns regarding delayed diagnosis. Many factors have been identified as contributing to the delay in presentation, including low awareness, a lack of routine screening, and the misconception that breast cancer primarily affects females [12]. Pulmonary metastases were the most common form of distant spread. In contrast, the most common site of a single metastasis reported from other studies is typically the bone, followed by the lungs [10]. However, bone metastases may be underreported in our study, as bone scans are not routinely performed in our settings.

Histologically, Invasive Ductal Carcinoma NOS was the predominant subtype (83.9%), consistent with findings reported in both male MBC and FBC populations [4]. In terms of molecular subtypes, Luminal A was the most common (61.8%), which aligns with the well-documented observation that MBC is frequently hormone receptor-positive. These findings are in agreement with previous studies [13]. However, the notable proportion of triple-negative breast cancer (TNBC) cases (20%) is of clinical significance, as this subtype is associated with a more aggressive disease course, poorer prognosis, and limited targeted treatment options. Moreover, the proportion of TNBC in MBC observed in this study closely mirrors that reported for FBC in the East African region, which ranges between 14.9% and 37.7% [14]. This similarity underscores potential regional or genetic factors influencing breast cancer subtypes in this population.

Despite the high prevalence of the Luminal A subtype, typically associated with favorable outcomes [15], our cohort of MBC patients experienced poor treatment results, with a five-year survival probability of only 11.6%, significantly lower than that of female counterparts receiving corresponding treatment strategies [16]. This may be attributed to a high proportion of patients presenting with locally advanced or distant disease, both recognized as poor prognostic indicators. Furthermore, most patients treated with curative intent were at stage III, and conventional imaging methods commonly used in our region, such as chest X-rays and abdominal ultrasounds, lack the sensitivity to detect small metastatic lesions [17], potentially leading to under-staging. Early recurrence, often within the first 15 months, was frequently observed among patients initially deemed candidates for curative treatment, further suggesting possible under-staging within this cohort [18]. However, no significant difference in mortality between sexes has been observed, indicating that tumor biology and behavior are the same in male and female patients [19].

In our study, the potential for poor treatment response among MBC patients remains uncertain due to limited data on chemotherapy and radiotherapy administration. Given the shared biological and histological characteristics of MBC and FBC, treatment strategies should be aligned to incorporate personalized care. Future studies in Tanzania should assess responses to chemotherapy and biological agents while also improving the detection of metastatic disease. Variability in the classification of malignant tumors (TNM) staging was evident, with primary tumor (T) status recorded in 38 of 56 patients, nodal involvement (N) in 32, and distant metastases in all. Despite 80% presenting with advanced disease, 90% underwent an MRM. In the era of de-escalating breast cancer surgery, these findings highlight the need to reconsider surgical approaches for male patients, as a less aggressive strategy may yield comparable or better outcomes.

## Conclusion

Most MBC patients presented with locally advanced or metastatic disease and experienced poor treatment outcomes, including low survival rates and disease progression. Despite the advanced nature of the disease in many cases, surgical intervention remained prevalent, prompting reflection on the balance between surgical management and disease stage. These findings highlight the urgent need for increased awareness, early detection programs, and improved access to diagnostic and treatment facilities for MBC in Tanzania. The prevalence of late-stage diagnosis suggests potential gaps in healthcare-seeking behavior, screening protocols, and referral systems. Furthermore, the apparent imbalance between the frequency of surgery and the advanced stage of the disease raises important questions regarding the role of neoadjuvant treatment and the potential for de-escalating surgical approaches in breast cancer. Future research should aim to address the key gaps identified in our study to optimize care strategies and enhance patient outcomes.

### What is known about this topic

- Male breast cancer is rare but more prevalent in sub-Saharan Africa;
- MBC patients present late with advanced disease, making treatment more challenging.

### What this study adds

- This study provides insights into the management of MBC in Tanzania;
- It highlights the treatment outcomes of MBC in resource-limited settings, including overall survival and disease-free survival, and also highlights areas that need further research.
